# Magnetic Nanoparticles from *Magnetospirillum gryphiswaldense* Increase the Efficacy of Thermotherapy in a Model of Colon Carcinoma

**DOI:** 10.1371/journal.pone.0108959

**Published:** 2014-10-07

**Authors:** Silvia Mannucci, Leonardo Ghin, Giamaica Conti, Stefano Tambalo, Alessandro Lascialfari, Tomas Orlando, Donatella Benati, Paolo Bernardi, Nico Betterle, Roberto Bassi, Pasquina Marzola, Andrea Sbarbati

**Affiliations:** 1 Department of Neurological and Movement Sciences, Human Anatomy and Histology Section, University of Verona, Verona, Italy; 2 Department of Biotechnology, University of Verona, Verona, Italy; 3 Consorzio Interuniversitario Nazionale per la Scienza e Tecnologia dei Materiali (INSTM), Firenze, Italy; 4 Department of Physics, University of Milano, Milano, Italy; 5 Department of Physics, University of Pavia, Pavia, Italy; 6 Department of Computer Science, University of Verona, Verona, Italy; Brandeis University, United States of America

## Abstract

Magnetic nanoparticles (MNPs) are capable of generate heating power under the influence of alternating magnetic fields (AMF); this behaviour recently opened new scenarios for advanced biomedical applications, mainly as new promising tumor therapies. In this paper we have tested magnetic nanoparticles called magnetosomes (MNs): a class of MNPs naturally produced by magnetotactic bacteria. We extracted MNs from *Magnetospirillum gryphiswaldense* strain MSR-1 and tested the interaction with cellular elements and anti-neoplastic activity both *in vitro* and *in vivo*, with the aim of developing new therapeutic approaches for neoplastic diseases. In *vitro* experiments performed on Human Colon Carcinoma HT-29 cell cultures demonstrated a strong uptake of MNs with no evident signs of cytotoxicity and revealed three phases in the interaction: adherence, transport and accumulation in Golgi vesicles. *In vivo* studies were performed on subcutaneous tumors in mice; in this model MNs are administered by direct injection in the tumor volume, then a protocol consisting of three exposures to an AMF rated at 187 kHz and 23kA/m is carried out on alternate days, over a week. Tumors were monitored by Magnetic Resonance Imaging (MRI) to obtain information about MNs distribution and possible tissue modifications induced by hyperthermia. Histological analysis showed fibrous and necrotic areas close to MNs injection sites in mice subjected to a complete thermotherapy protocol. These results, although concerning a specific tumor model, could be useful to further investigate the feasibility and efficacy of protocols based on MFH. Magnetic nanoparticles naturally produced and extracted from bacteria seem to be promising candidates for theranostic applications in cancer therapy.

## Introduction

Thermotherapy represents an effective tool for the treatment of many types of tumor but it is strongly hampered by poor specificity of the induced lesion [1], [2], [3]. Several approaches have been proposed to improve the efficacy of the technique: one of them is based on intratumoral delivery of magnetic nanoparticles (MNPs) followed by application of alternating magnetic fields (AMF) to increase the local temperature of the tissue thus the effectiveness of the method [3], [4], [5]. This approach is called magnetic fluid hyperthermia (MFH).

In 1963, the Italian scientist Salvatore Bellini reported the first description of magnetotactic bacteria. This subtype of organisms naturally produces magnetic iron-oxide nanoparticles and uses them as a compass for geomagnetic navigation to search for optimal growth conditions [6], [7]; those nanoparticles have been over time referred as magnetosomes (MNs). A more complete description of magnetotactic bacteria was later reported [8].

Recently, MNs have been proposed as active agents in hyperthermia for cancer therapy [9], [10]; moreover, MNs extracted from magnetotactic bacteria [11], [12] are considered with increasing interest as therapeutic and diagnostic agents mainly because of their physical properties. It has been reported that, for an applied AMF of frequency and strength kept below the tolerance threshold limit of about 200 kHz and 100 mT respectively, the specific absorption rate (SAR) of the chemically synthesized nanoparticles, currently used in magnetic thermotherapy, is several orders of magnitude lower than the one of the biologically synthesized magnetosomes [9], [10]-[13]. Due to their magnetic properties, MNs also show high transversal relaxivity [14], a feature that makes these MNPs highly efficient contrast agents for magnetic resonance imaging (MRI).

The bacterial strain considered in the present investigation, *M. gryphiswaldense*, is able to internalize iron in ionic state from the environment and to synthesize iron oxide nanoparticles (size range 35-50 nm) of cubic-octahedral shape [15], [16]. This biological process is extremely repeatable with a very low variance in terms of size and shape of resulting nanoparticles.

As mentioned before, MNs isolated from magnetotactic bacteria develop a high heating effect when exposed to AMF. Preliminary studies prove the possibility of using these MNs as therapeutic agents when delivered in tumors [4], [10].

However, data on the therapeutic effect and biological interaction of MNs with living tissues are still scarce, and their toxicity has not been definitively assessed [17]; moreover, ultrastructural studies on the localization of nanoparticles in the intracellular compartments are absent [16]-[18].

In the present study, the interaction of MNs extracted from M. *gryphiswaldense* with tumor cells was investigated by using Human Colon Carcinoma cells (HT-29). Hyperthermia treatment of colorectal cancer is of particular interest for potential clinical applications: for this purpose we used a protocol that include molecular biology, histology, electron microscopy analysis and magnetic resonance imaging.

The interaction of MNs with tumor cells and their therapeutic potential was also investigated *in vivo* in nude mice. In order to assess the potential usefulness of MNs in magnetic thermotherapy, we monitored, by MRI an experimental model of colon carcinoma before and after the treatment with thermotherapy.

## Materials and Methods

### *In vivo* experiments: experimental design and thermotherapy protocol

Twenty-two nude homozygote male mice (Harlan Laboratories, Udine, Italy) were maintained under standard environmental conditions (temperature, humidity, 12 h/12 h light/dark cycle, with water and food ad libitum) under veterinarian assistance. Animals handling and surgery were performed following a protocol approved by the Animal Care and Use Committee of the University of Verona (CIRSAL), and by the Italian Ministry of Health, in strict adherence to the European Communities Council (86/609/EEC) directives, minimizing the number of animals used and avoiding their suffering.

In a preliminary study on a limited number of animals, one million HT-29 cells, resuspended in 200 µl of sterile PBS, were subcutaneously injected in the right flank of anesthetized mice. Ten days after inoculation of tumor cells, mice were monitored by MRI to measure the tumor size: animals whose tumor volumes ranged between 0.4 and 0.5 cm^3^ were injected with MNs. We observed that usually it was possible to inject a total volume of about 200 µl by using 3 to 4 separate punctures without relevant leakage of fluid from the tumor mass. These findings were then used to design the experimental protocol.

A population of n = 22 mice was divided in two groups: control and experimental. The control group (n = 12) was further divided in three subgroups: the first subgroup (n = 4) was administered with 1 mg of MNs diluted in 200 µl of PBS and did not receive AMF exposure. Administration of MNs was performed by direct injection into the tumor mass using an intradermal needle (21G). The second subgroup (n = 4) was exposed to AMF without MNs administration. The third subgroup (n = 4) did receive neither thermotherapy nor MNs administration.

The experimental group (n = 10) received 1 mg of MNs diluted in 200 µl of PBS and was exposed to AMF on day 0 (immediately after injection), 2 and 4. The AMF apparatus yield a magnetic field of intensity 23 kA/m (∼29 mT) with a frequency of 187 kHz. These values are very close to those used in human application of hyperthermia [19]. Mice were placed on a custom made animal holder under isofluorane anesthesia 1.5% (Forane, Abbott) and exposed to AMF for 20 min. A digital IR camera (Flir I7, Flir Systems, Italy) was used by the operator to record maps of heating distribution in the tumor and surrounding tissues.

All the mice were monitored by MRI at three time points: 24 h, one week and two weeks after MNs administration. The first and second points were chosen to evaluate the physiological distribution of MNs and acute effects induced by MFH inside the neoplastic mass, respectively. The last point was useful to check whether some kinds of alterations do occur after the treatment. Animals were then sacrificed and tumors excised for histological analysis. Three mice died during the first steps of the treatment, probably due to an overdose of anesthetic. Mice were sacrificed by isofluorane overdose and successively by neck dislocation. These methods were performed in according to D.L. 4 March 2014 n°26 of the Italian Ministry of Health.

### *Magnetospirillum gryphiswaldense* culture

*M. gryphiswaldense* MSR-1 (DSM6361) purchased from Deutsche Sammlung von Mikroorganismen und Zellkulturen GmbH (Germany) was cultured in specific growth medium (optimized during the present study) containing 0.1 g KH_2_PO_4_, 0.15 g MgSO_4_*7H_2_O, 2.38 g HEPES, 0.34 g NaNO_3_, 0.1 g yeast extract, 3 g soy bean peptone, and 1 ml EDTA-chelated trace element mixture. The medium contained also 27 mM potassium l-lactate as carbon source and 100 µM of ferric citrate as iron source [20]. A single colony of MSR-1 from agar plates was transferred to a tube containing 10 ml of liquid medium and grown with 100 rpm shaking at 28°C for 24 h. This inoculum was grown by four escalating volume transfers at a ratio of 10% (v/v) before being used as inoculum in a 5 L screw cap bottle culture. Culture was maintained at 28°C and agitated at 100 rpm for 24 h.

### Purification of MNs

The purification of MNs was performed according to the protocol proposed by Grunberg [20] as follows: 1) lyses of 10 g of *M. gryphiswaldense* dry weight with 50 ml of 50 mM HEPES 4 mM EDTA (pH 7.4) using French-press (1.26 Kbar); 2) centrifugation at 680 g for 5 min to remove non lysated materials; 3) magnetic separation with magnetic column (MACS, Miltenyi Biotec); 4) MNs were eluted with 10 mM HEPES (pH 7.4), positioned on a scaffold of sucrose (55% w/w in 10 mM HEPES), MNs were centrifuged at 280,000 g for 12 h at 4°C and the pellet was incubated with 10 mM HEPES for 16 h at 4°C to allow for solubilization. After purification, MNs were dried for 5 h using a lyophilizer, irradiated with γ-rays (56 Gy for 84 min) and finally stored at -20°C.

### Transmission electron microscopy (TEM)

Whole mount bacteria of different samples of MNs, extracted and purified, were fixed with glutharaldehyde 2% in Sorensen buffer pH 7.4 for 2 h, post-fixed in 1% osmium tetroxide in aqueous solution for 2 h, dehydrated in graded concentrations of acetone. At the end of dehydrating process, samples were positioned in a multi-well grid for electron microscopy and observed using an EM10 electron microscope (Zeiss, Oberkocken, Germany).

### Scanning electron microscopy (SEM)

Specimens were fixed with glutharaldehyde 2% in 0.1 M buffered phosphate, dehydrated in graded ethanol, critical point dried (CPD 030, Balzers, Vaduz, Liechtenstein), fixed to stubs with carbon-based adhesive, sputtered with carbon by MED 010 coater (Balzers), and examined with an XL30 ESEM scanning electron microscope (FEI Company, Eindhoven, Netherlands) equipped for Energy Dispersion Analysis of X-Ray (EDAX).

### Dynamic light scattering (DLS)

Hydrodynamic radius of MNs was determined from a batch of 0,5 mg resuspended in 5 ml of HEPES 10 mM, ultrasonicated for 10 min and filtered using a 450 nm filter. DLS data were obtained using a He-NE 633 nm wavelength laser with a goniometric set of 173° (Malvern Instrument LTD, UK), normalized to unity and reported in logarithmic scale.

### Susceptibility measurements

Magnetic measurements were carried out on powders (total MNs weight  = 3.6 mg) using a Quantum Design SQUID MPMS XL-7 magnetometer. The zero field-cooled and field-cooled (ZFC/FC) magnetization curves were obtained for different applied magnetic fields in the temperature range 2–300 K, while the field dependent magnetization measurements were recorded in the range of ±5 T at both 2 K and room temperature.

### Thermal properties of MNs

A sample containing 6.7 mg of purified, sterilized and lyophilized MNs was exposed to an AMF (187 kHz, 23 KA/m [∼29 mT]) for 24 min. The increment of temperature was measured by an infrared camera Flir i7 (Flir System Inc., Italy). MNs were then diluted in distilled water at concentration of: 3 mg/ml, 2 mg/ml, 1 mg/ml, 0.5 mg/ml and exposed to AMF using the same frequency and field strength. In order to estimate the iron content of our nanoparticles, 4 mg of extracted MNs were dispersed in a 1∶3 mixture of H_2_SO_4_ and HNO_3_ and digested using a microwave digestor. The preparation was analyzed by Atomic Adsorption Spectroscopy (AAS, Perkin-Elmer 1100B).

### Cancer cells culture

Human Colon Carcinoma cells (HT-29) purchased by ATCC (Manassas, VA), were cultured in Dulbecco's Modified Eagle Medium (DMEM) with 10% of Fetal Bovine Serum (FBS) and 1% of a mix of penicillin/streptomycin 1∶1, in 25 cm^2^ plates and incubated at 37°C in humidified air with 5% CO_2_. Medium, serum and antibiotic mix were purchased by GIBCO, Life Technologies, USA. When at confluence, cells were treated with trypsin-EDTA 1% (GIBCO, Life Technologies, USA), harvested and centrifuged at 1200 rpm for 5 min. The supernatant was discarded and cells pellet was resuspended in 1 ml of complete medium, placed in 75 cm^2^ plates and incubated at 37°C and 5% of CO_2_ until 80% confluence was detectable.

### *In vitro* MTT test

The cytotoxicity of MNs versus HT-29 cells was assessed by the loss of cells viability using 3-(4,5-dimethylthiazol-2-yl)-2,5-diphenyltetrazolium bromide) (MTT) test. Cells were plated at a density of 8*10^3^ cells per well in 96-well plates and incubated at 37°C in a mixture of air and 5% CO_2_. After 24 h, the medium was replaced with fresh medium containing 1, 0.5 and 0.2 mg/ml of sterilized MNs, respectively. After 6, 12 and 24 h of incubation, 10 µl of MTT solution (Sigma, Italy) was added to each well. Plates were then incubated for additional 4 h (37°C, 5% CO_2_). After the incubation time, plates were removed from the incubator and formazan crystals formed by the living cells were dissolved in 100 µl of dimethyl sulfoxide (Sigma, Italy). The multiwell was placed into a microplate reader (CHROMATE 4300 Awareness Technology, USA) for the measurement of absorbance at 570 and 630 nm. Four measurements of optical density (OD) were recorded for each sample and cell viability (%) was calculated with the following equation: CV% = (OD_sample_/OD_control_) • 100.

### *In vitro* assay of MNs-uptake in cancer cells

A plate containing 2*10^5^ cells was placed in specific glasses multiwell for optical microscopy (Falcon BD, Italy) with 1 ml of growth medium. Cells were incubated at 37°C in humidified air with 5% CO_2_ for 24 h. The growth medium was then discarded and replaced with fresh medium added with MNs at different concentrations: 1 mg/ml, 0.5 mg/ml, 0.2 mg/ml. Cells were incubated as previously described for 6 h, 12 h and 24 h. At each time point, the growth medium was discarded and cells were washed with 1 ml of 1X sterile phosphate buffer saline (PBS, GIBCO, Life Technologies, USA). Cells were fixed with 1 ml of 4% buffered formalin (Bioptica, Italy) for 30 min at room temperature. Once formalin was discarded, cells were double stained: Prussian Blue to visualize MNs and Nuclear Fast Red (Bioptica, Italy) to visualize nuclei. HT-29 samples were observed at 10X, 20X and 40X optical magnification using an Olympus microscope (BX-URA2, Olympus optical, GMBH, Hamburg, Germany) equipped with Image ProPlus software (Media Cybernetics, Rockville, USA).

### TEM of cells incubated with MNs

HT-29 cancer cells were plated on a 2.4 cm culture glass, positioned on the bottom of 3.5 cm Petri dishes and incubated at 37°C in humidified air with 5% of CO_2_ in 3 ml of growth medium. After 24 h, cells were fixed with glutaraldehyde 2% in Sorensen buffer pH 7.4 for 2 h, then post-fixed in 1% osmium tetroxide (OsO_4_) in aqueous solution for 2 h, and finally dehydrated in graded concentrations of acetone. At the end of dehydrating process, glasses were stained with lead citrate and observed using a XL30 ESEM scanning electron microscope (FEI Company, Eindhoven, Netherlands).

### MRI

MR images were acquired in order to monitor the tumor growth at days 1, 7 and 14 both for control and experimental groups. MRI was performed using a Bruker tomograph operating at 4.7 T, equipped with an actively shielded gradient insert (Bruker, Germany) having a maximum gradient strength of 40 G/cm. Animals were placed prone in a heated bed and a 3.5 cm i.d. birdcage coil was used to acquire the MR signal. T_2_ and T_2_* weighted images were acquired to detect the tumor and the presence of MNs respectively. T_2_weighted images were acquired using a RARE 3D sequence with TR  = 1200 ms, TE_eff_ = 47.5 ms, FOV = 5×2.5×2.5 cm^3^, NEX  = 1, MTX  = 256/128/32, Slice Thickness  = 0.78 mm. T_2_*weighted images were acquired using a FLASH gradient echo sequence with TR  = 400 ms, TE  = 4.4ms, flip angle  = 10°, FOV  = 5×2.5 cm, NEX  = 2, MTX  = 256/128, NSLICES  = 8, Slice Thickness  = 2 mm.

### Histology

After the last MRI acquisition, animals were sacrificed and tumors were excised, washed with PBS 0.1 M and fixed with 10% buffered formalin for 4 h. Afterwards, samples were dehydrated with increasing concentration gradient of alcohol from 70% to 100% and then with xylene for final processing. Tissues were embedded in paraffin and sections of 5 µm were obtained and dried at 37°C for 24 h. Sections were stained with Prussian blue and Nuclear Fast Red (Bioptica) to visualize iron nanoparticles and nuclei respectively.

## Results

### Morphology and physical properties of MNs

**Figure 1:**
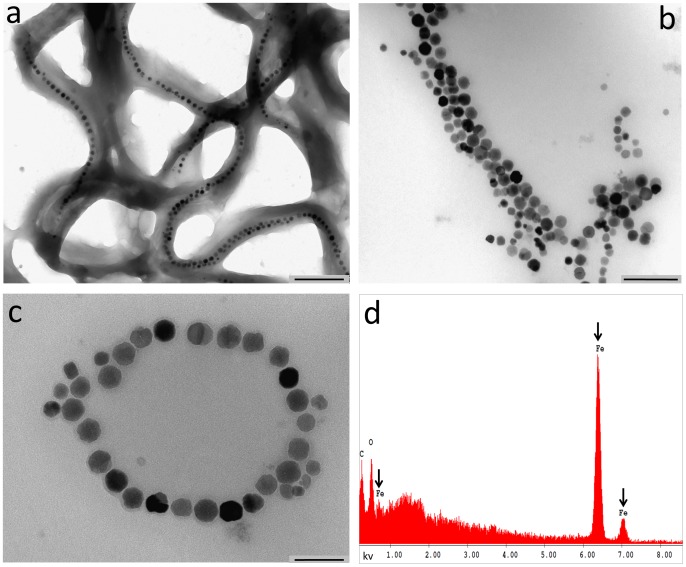
TEM of MNs. Panel a shows the organization of MNs in chains in the bacteria (scale bar, 500 nm). Panels b-c show that the typical conformation of chains is maintained after isolation of MNs. Scale bars: b>200 nm, c>100 nm. Panel d, X-ray microanalysis shows the iron content of the MNs.

TEM of whole-mount bacteria, observed by backscattered electron detector, showed that MNs are organized in long chains (**Figure 1a**) along the major axis of each bacterium. **Figure 1b and 1c** show TEM images of MNs extracted from M. *gryphiswaldense*. The cubic-octahedral structure of crystals (mean Feret's diameter of iron core  = 42±9 nm) and the surrounding membrane (see arrows in **Figure 1c**) are clearly visible. AAS reported an iron content 0.167 mg Fe/g of extracted MNs, EDAX spectrum showed the absence of any metal except iron (**Figure 1d**).

**Figure 2:**
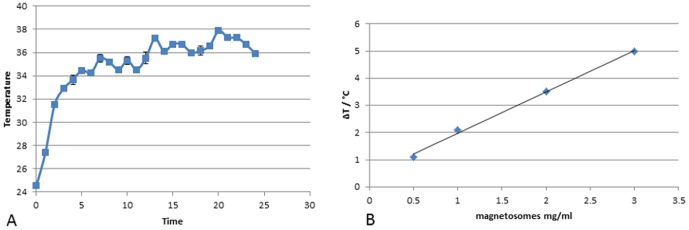
Thermal properties of MNs in alternate magnetic field. Variation of temperature of samples containing: (a) 6.7 mg of MNs lyophilized and (b) MNs diluted in distilled water at concentration of: 3 mg/ml, 2 mg/ml, 1 mg/ml, 0.5 mg/ml exposed to an AMF of 187 kHz (23kA/m) as a function of time, measured by infrared camera.

A temperature increment of about 10°C was obtained in lyophilized MNs sample (6.7 mg) upon application of AMF for 24 min (**Figure 2a**). The increment of temperature consistently decreased with the concentration of MNs diluted in distilled water. Such enhancement amounted to about 5°C for a sample containing 3 mg/ml of MNs, to about 3°C for the sample containing 2 mg/ml and to about 2°C for the sample containing 1 mg/ml of MNs. The less concentrated sample, with 0.5 mg/ml of MNs, gained an increment of 1°C (**Figure 2b**).

**Figure 3:**
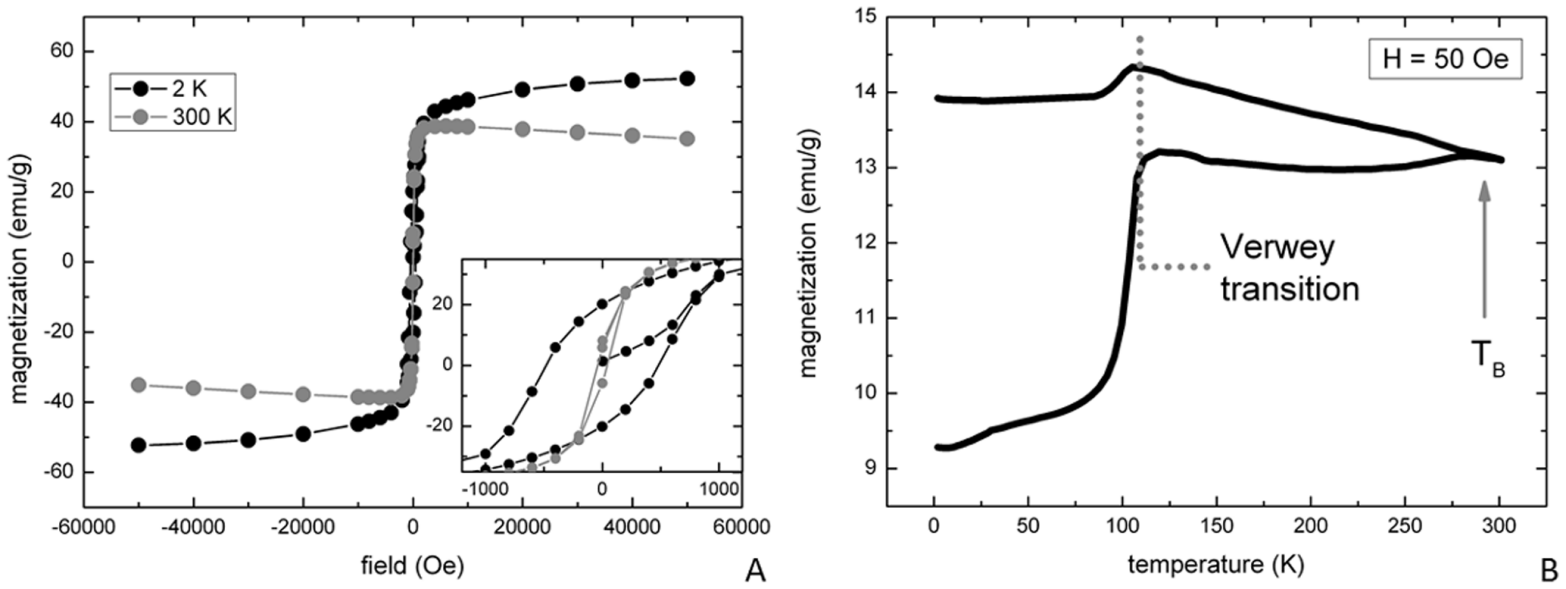
Susceptibility measurement. DC susceptibility measurements performed on a freeze-dried magnetosomes sample: (a) hysteresis loops at high (300 K) and low (2 K) temperature and (b) Zero Field-Cooled/Field-Cooled (ZFC/FC) curves collected at low field, H = 50 Oe.

The magnetic measurements of MNs revealed, as expected, a single-domain superparamagnetic-like behaviour with almost closed hysteresis at room temperature (**Figure 3a**) and irreversibility effect in ZFC/FC curves. The saturation magnetization, Ms ∼40 emu/g, is compatible with values commonly reported for magnetosomes [13] and iron oxide nanoparticles in general. From the ZFC curve maximum displayed at low applied magnetic field (**Figure 3b**), the superparamagnetic blocking temperature is estimated to be T_B_ ∼300 K or higher. In addition, a discontinuity in the magnetization curves as a function of temperature can be clearly seen at around 110 K (**Figure 3b**). This is possibly an evidence of the Verwey transition, a long range charge re-arrangement that occurs mainly in transition metal oxides; such transition is also a signature for the existence of magnetite phase [21].

### DLS

**Figure 4:**
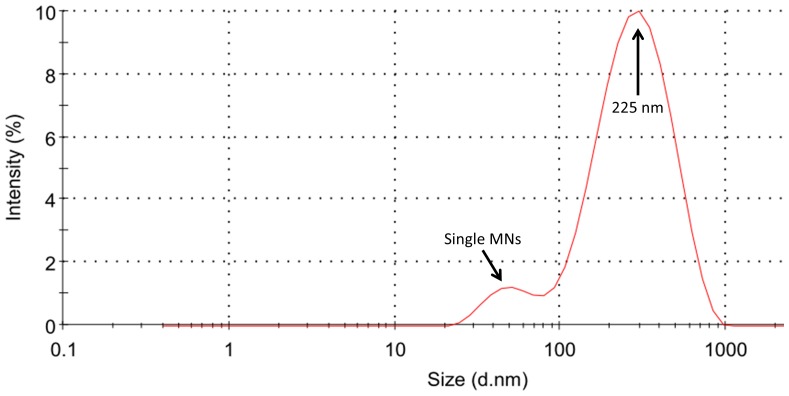
Dynamic Ligth Scattering. Scattered light intensity weighted magnetosomes size distribution in ultrasonicated and filtered HEPES solution; the two peaks centered at 225 nm and 40 nm are in good agreement with the size of chains of magnetosomes and single nanoparticles, respectively.

The distribution of the mean hydrodynamic radius shows a peak centered at 225 nm; this value is in good agreement with other data obtained on magnetosomes [22], [23]. A second peak, centered around 40 nm can be attributed to small single nanoparticles (**Figure 4**).

### *In vitro* uptake of MNs in HT-29 cancer cells

**Figure 5:**
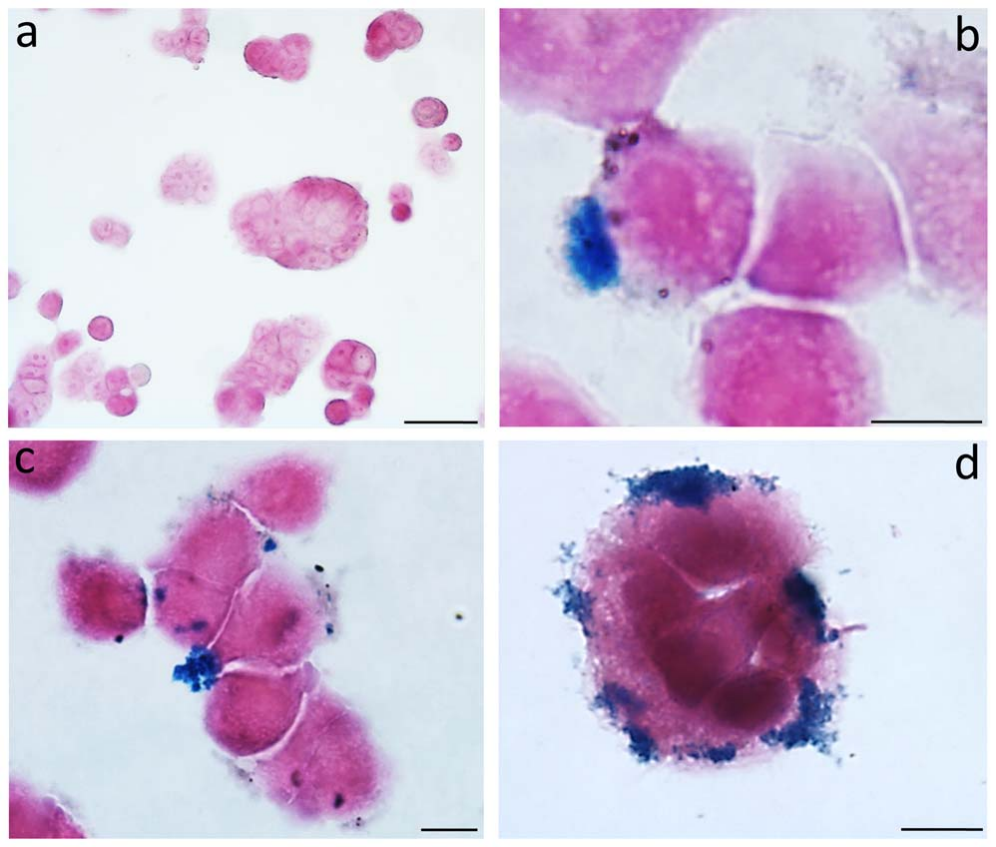
Cancer cell culture. The figure shows the untreated cells (a) and MNs-treated cells (b-d) observed after 24 h. Prussian Blue evidences the iron depots. Enlargement: a>50 µm, b>10 µm, c>10 µm and d>10 µm.

Prussian Blue staining demonstrated a strong uptake of iron nanoparticles by HT-29 cells as clearly depicted in **Figure 5**. MNs were detected primarily on the cell membrane although some were also visible within cytoplasm. The effect of varying MNs concentration in the culture medium and incubation time was investigated. Qualitative analysis of stained samples showed that, among the different concentrations tested (ranging from 0.2 to 1 mg/ml), the best condition for a good internalization was 0.2 mg/ml of MNs; the most effective time of incubation, with purified and sterilized MNs, was 24 h. Despite the high level of iron internalization, detectable changes of phenotype were not appreciable. (**Figure 5b-d**).

**Figure 6:**
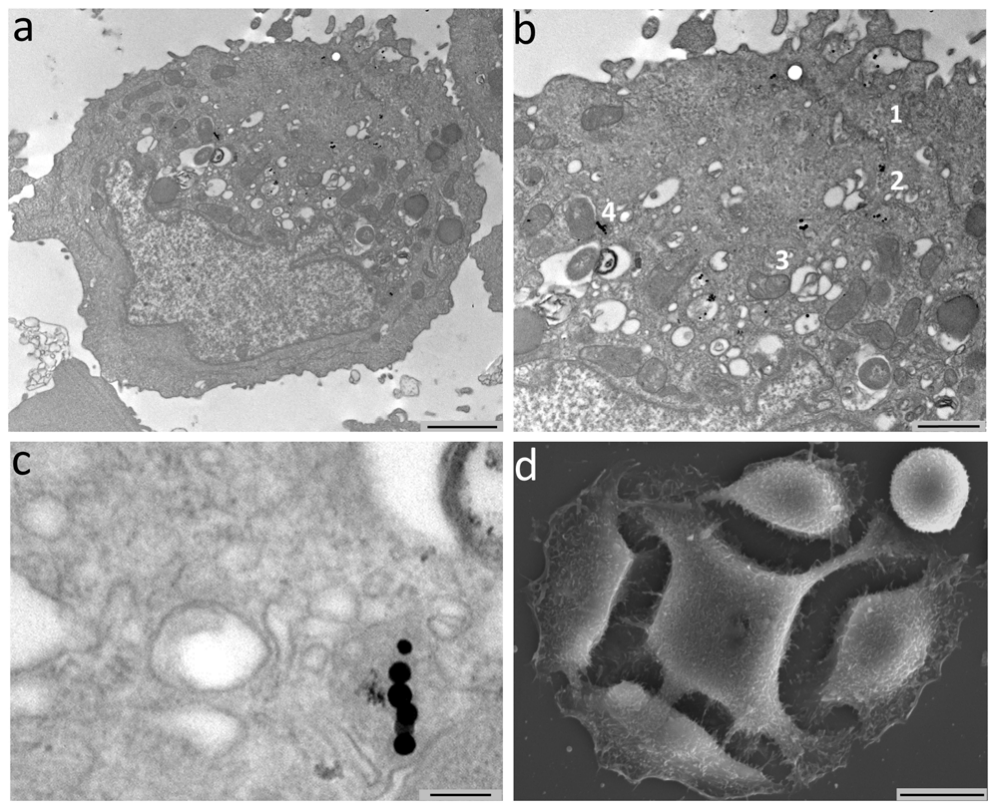
TEM shows the internalization of chains of MNs in HT-29 cancer cells. The chains that have penetrated in the cells are composed by 6-10 units of MNs and are positioned near the nucleus (Panel a). In Panel b, MNs are visible at cell membrane (arrows) or in the Golgi complex. Panel c shows the localization of the MNs in cytoplasmic vacuoles at high enlargement (Scale bars, a 2 µm, b 1 µm, c 120 nm, d 10 µm). Panel d shows a representative SEM image of HT-29 cancer cell (0.2 mg/ml MNs 12 h); no appreciable alterations of the surface are visible when compared to controls.

The internalization process of MNs was deeply investigated by TEM: representative images are reported in **Figure 6**. At least three kinds of interaction were found, likely corresponding to three different internalization steps. In the first step MNs were adherent to the cell membrane, with the formation of invaginations surrounding MNs chains (**Figure6 1b**). In the second step (**Figure 6** **2b**), MNs were located into the cytoplasm, enclosed in vesicles. In the third step (**Figure 6** **3b**), MNs were found into the Golgi apparatus and specifically into the innermost vesicles. In each of these steps, MNs were detectable as isolated particles, as small groups or short chains (**Figure 6** **a,b**). MNs were found in vesicular formations similar to lysosomes presumably excreted from the Golgi apparatus. In some cases, these formations had a multi-vesicular aspect, while in others they appeared filled with material similar to multilamellar phospholipids. TEM images performed on HT-29 cancer cells showed the presence of chains of MNs in the cytoplasm, confirming the internalization process and the stability of the chain structure (**Figure 6c**).

SEM images did not show any appreciable alteration of the cellular surface in comparison to the controls (**Figure 6d**).

### MTT test

**Figure 7:**
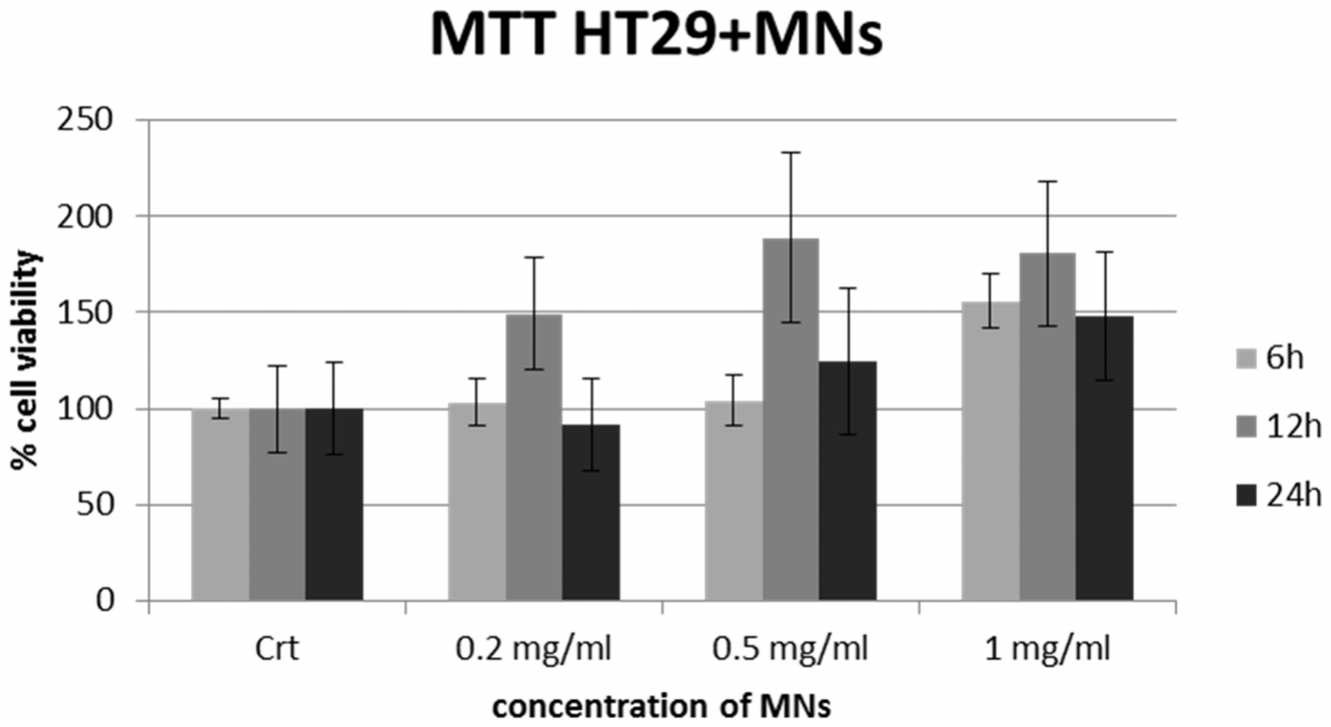
MTT assay on HT-29 cells after uptake with MNs. MTT assay shows a negligible cytotoxic effect of MNs: percent viability of cells incubated with MNs is expressed relevant to control cells.

MTT assay revealed that different amounts of sterilized MNs (1 mg/ml, 0.5 mg/ml, 0.2 mg/ml), when incubated for varying time periods with HT-29 cells, do not show statistically significant negative effects (one way ANOVA, p>0.05) on cell viability at any dosage. The results are reported in **Figure 7**.

### *In vivo* experiments

Thermal images were acquired in each mouse during the exposure to AMF by using an infrared camera. Temperature increments ranging between 2 and 3°C were observed in tumors treated with MNs. On the other side, negligible variations of thermal maps were recorded in tumors exposed to AMF that did not receive MNs injection.

**Figure 8:**
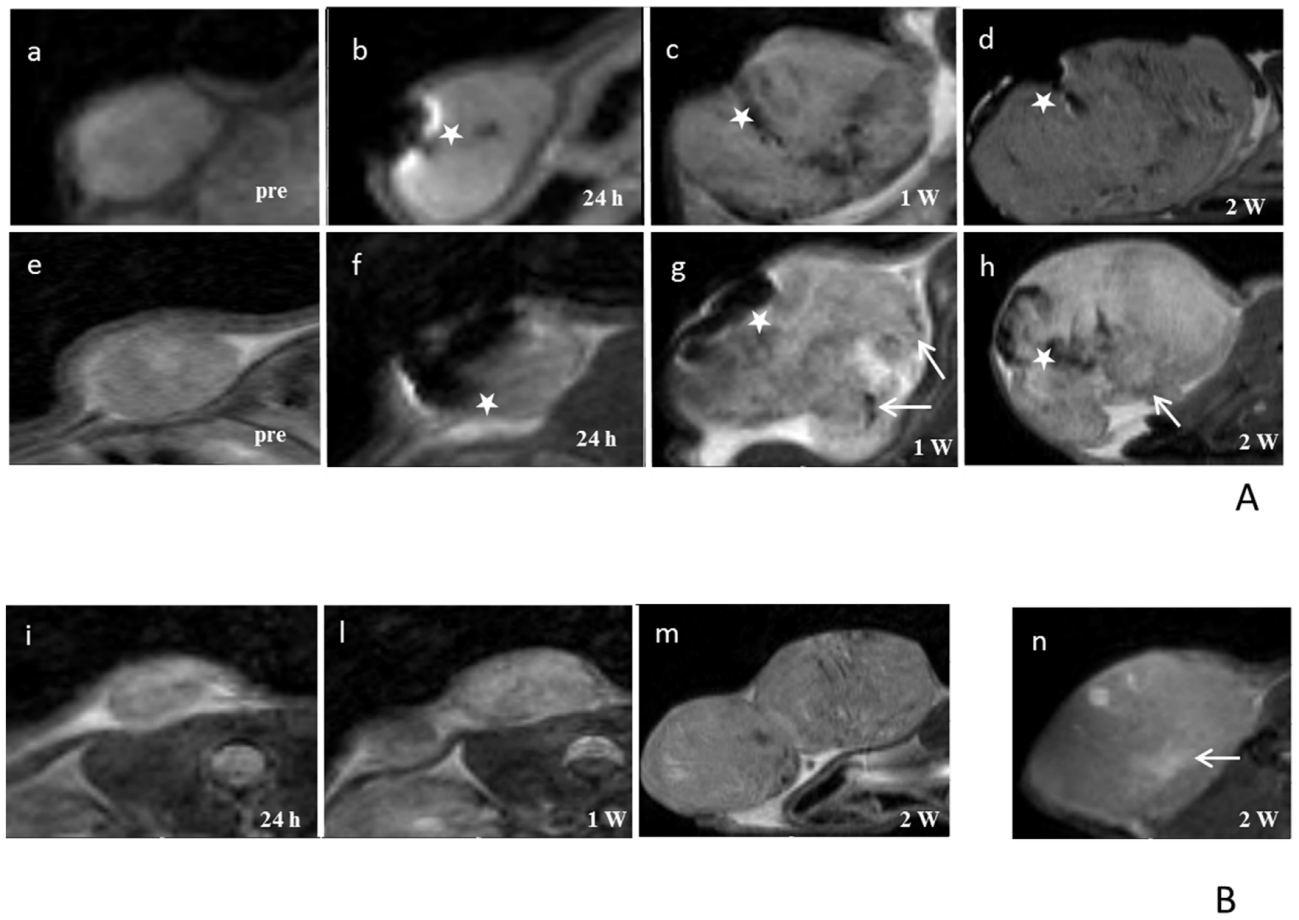
MR Images of representative animals. A) MRI Upper line: animal treated with MNs injection; images acquired before MNs injection (a), 24 h (b), one week (c) and two weeks (d) after MNs injection. Second line: animal treated with MNs injection with AMF; images acquired before MNs injection (e), 24 h (f), one week (g) and two weeks (h) after MNs injection. Magnetosomes are injected in tumor mass and MRI allows the detection of injection sites (white stars). B) animal treated with AMF; images acquired before 24 h (i), one week (l) and two weeks (m) after treatment. Control animal not treated with MNs and AMF; images acquired after two weeks (n).

MR images were acquired to monitor the development of tumor mass before and after hyperthermia treatment: as reported in **Figure 8**, several regions of strong signal drop are clearly visible. In T_2_-weighted images, most of the neoplastic parenchyma was iso-intense. In the first subgroup of control group (**Figure 8A** **a-d, white stars**), the injection of MNs was clearly detectable and MRI showed areas of signal drop due to the presence of iron in the injection site. No other relevant changes were detectable in the signal intensity of the tumor parenchyma. This strong, negative enhancement, common to iron-based nanoparticles [24], [25], sets the basis for potential application of MNs as negative contrast agents in MRI. This feature has relevance in the possible translation of these methods to the clinics for theranostics applications. Mice in the second subgroup of control group (**Figure 8B** **i-m**) showed moderate increase of signal intensity in small regions of the tumor, probably due to edema associated to necrosis caused by fast tumor growth. In the third subgroup of the control group (**Figure 8B** **n**), tumor appeared as a well defined mass with sharp boundaries, located in subcutaneous tissue (see arrow). Furthermore, a thin hyperintense layer was detectable at the edges of the tumor (**Figure 8B** **n**). Areas of high intensity were detectable and attributable to edema. In the central portion of the tumor, areas of low signal intensity, that could be identified as necrotic or hemorrhagic sites, were observable (see arrow). In the experimental group (**Figure 8A** **e-h**), tumors appeared as non-homogeneous masses characterized by regions of moderate loss of signal (probably corresponding to necrosis, see arrows), while areas with a higher loss of signal represent regions of MNs diffusion (white stars in **Figure 8A** **g, h**). Compared to the control group (third subgroup, **Figure 8B** **n**), there was an increment of both the edematous (hyperintense) and necrotic/hemorrhagic (hypointense) components. In some cases, these degenerative aspects were prominent in correspondence to the injection site.

MR images performed after a complete thermotherapy protocol (**Figure 8A** **g** ), did not show significant differences in signal intensity when compared to images acquired 24 h after MNs injection (white stars, **Figure 8A** **f**).

### Histology

**Figure 9:**
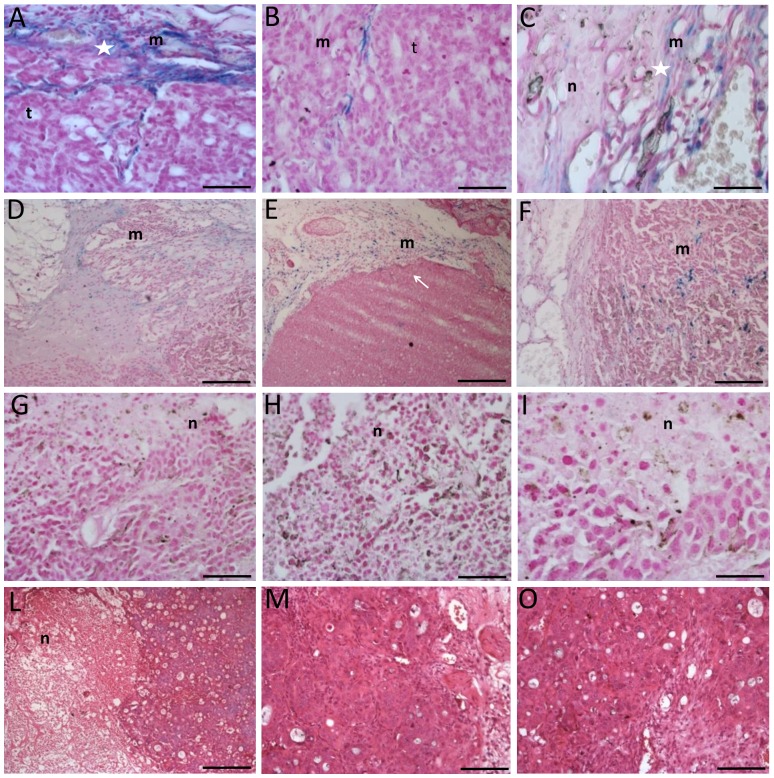
Histological analysis of tumors. In the Panel A-C histology of tumor of the experimental group is showed. Injection site is showed in Panel A while in Panel B the living tumor area is illustrated. In the Panel C injured tumor area is showed. In Panel E-F histology of first subgroup of the control group is presented. In Panel G-I histology of second subgroup of the control group is presented and in the Panel L-N is showed the third subgroup of the control group. The presence of MNs depots is detectable in injection sites (A). MNs are capable to migrate and spread in tumor tissue causing the formation of fibrotic and necrotic areas (B, C). Scale bars, A-C 60 µm, D-E 300 µm, F and G 120 µm, H-I 60 µm, I 300 µm, M 120 µm, N 60 µm. Legend: m = MNs, n =  necrosis and t  =  tumor.

Histological examinations contributed in elucidating the effect of different treatments. Sections obtained from tumors treated with MNs and thermotherapy showed clear evidence of edematous and necrotic phenomena that were not evident in corresponding sections extracted from the control group (**Figure 9** **A-C**). The injection site of MNs was clearly visible (white star, **Figure 9A**) and is characterized by degenerating unclustered cells intruded by extracellular matrix. Evidences of necrosis and fibrosis, induced by temperature enhancement, were significantly spread and detectable also in regions away from the site of injection (white star, **Figure 9C**). Tumors treated only with MNs (first subgroup, **Figure 9** **D-F**) did not show appreciable differences compared to those of the second subgroup; in these tumors the site of injection was highlighted by histochemical staining. Iron nanoparticles marked with Prussian Blue staining were detected in subcutaneous areas and within the tumor mainly along the connective tissue septa or in perivascular locations. There, elongated cellular elements showing a marked uptake of MNs were present and possibly identifiable as macrophages (**Figure 9** **, D-F**). Tumors belonging to the third subgroup (**Figure 9** **, L-N**) were characterized by the presence of a thin connective capsule surrounded by a layer of adipose tissue, with some connective branches infiltrating the neoplastic mass. Tumor tissue showed the typical features of epithelial tumors: a solid mass with a high nucleo-cytoplasmatic ratio arranged in nests. Isolated areas of edema, necrosis or hemorrhage were visible usually in the central portion of the histological sample. Tumors of the second subgroup did not shown marked differences compared to those of the third subgroup with the exception of a moderate increase in edematous or necrotic components (**Figure 9** **, G-I**).

Concerning the persistence of the MNs, histological examinations performed two weeks after administration revealed the presence of MNPs next to the injection site, with spots also detectable in the surrounding parenchyma.

## Discussion

A renewed interest in biomedical applications of MNPs has increased considerably over the past years, with MNs attracting widespread attention because of their prospective medical applications either as contrast agents for MRI, as heating mediators for cancer therapy by intracellular hyperthermia or as drug delivery carrier [14], [26], [27], [28]. The efficiency of MNs as heating mediators is strongly dependent on the interaction with cells and the capability to cross the phospholipid membrane [29].

Recent studies on cancer proposed a new potential role of MNs as “theranostics” agents. The therapeutic efficacy of MNPs on mouse mammary carcinoma, glioblastoma, and prostate cancer demonstrated the feasibility and efficacy of this heating method, as well as a very low clearance rate of these nanoparticles from tumors, allowing for longitudinal treatment following a single magnetic fluid injection [3], [30]. MNPs have been used in clinical trials with prostate cancer [4] and glioblastoma multiforme [2]. Although these studies proved that thermotherapy of tumors using MNPs can be safely applied, studies on the biological interaction between cells and MNPs are still poor.

The aim of this study was to analyze the interaction, both *in vitro* and *in vivo*, of tumor cells with MNs extracted from a strain of magnetotactic bacteria. Among different strains, we chose *M. gryphiswaldense* for its availability and lack of knowledge about its biological interaction with cells. TEM images on bacteria and isolated nanoparticles confirmed the cubic-octahedric shape and the organization in chains; DLS data, obtained on an homogeneous solution, showed the presence of clusters of about five elements along with a small number of single MNs. Some researches focused on chains length suggesting a relation between this parameter and the thermal properties of MNs. Our findings on ultrastructural analysis show that even though MNs extracted from bacteria are actually organized in chains of several elements as previously reported [10], they enter the intracellular space as single nanoparticles or as short chains.

Due to intrinsic limitations of standard histology, the interaction between MNs and tumor cells was investigated at a higher spatial resolution by electron microscopy. TEM images suggested a determined multistep pathway of MNs internalization. After adhesion to the phospholipid membrane, MNs were enclosed in vesicles, carried near the Golgi apparatus and finally included in vesicles that resemble a double layered membrane.

It is well known the high sensitivity of tumor cells to thermal variation. Temperatures ranging between 42 and 47°C degrees cause degenerative processes in neoplastic cells [25]. We tested thermal properties of MNs from *M. gryphiswaldense* included in a tumor mass with a protocol based on hyperthermia induced by an AMF and the reaction of the xenograft neoplasm was observed by MRI and histology. Contrary to what previously reported [9], in none of the animals belonging to the experimental group a complete remission of the tumor after the treatment was obtained. The lower AMF strength and frequency (∼29 mT vs. 40 mT, 187 kHz vs. 183 kHz) as well as intrinsic magnetic properties of MSR-1 MNs, could be hypothesized as sources of differences against the results described in the literature.

However, we showed a local effect of MNs within tumors, with evidences of tissue necrosis both by histology and MRI (**Figures 8** **-** **9**). Furthermore, longitudinal MRI data demonstrated that MNs spread from the injection site through the surrounding tissue (**Figure 8A** **g, h**).

The physiological conditions for MNs migration still need to be clarified; a possible scenario involves the combination of passive transport mediated by reticuloendothelial system and active transport, mediated by macrophages. These processes resemble the transport pathways for conventional iron oxide nanoparticles, as described in [31].

The proposed experimental design, that involves both *in vitro* and *in vivo* examination, allows for non invasive follow up of the lesions at different time points. In addition, our results demonstrate that clinical MRI could be included in protocols based on theranostic employment of MNs: both the injection site and the necrotic areas of the tumor can be detected at once in a single MRI session. Theranostic is a combination of diagnostics and therapy and our data confirms that MNs exhibit some core characteristics of the so called “theranostic agents”.

Our results do not match data available in the literature; it has to be noticed that different parameters used for hyperthermia, as well as different strain of magnetotactic bacteria used to obtain MNs and a different tumor model, are all features that could lead to some inconsistencies with previous discoveries. Future efforts will focus on the optimization of experimental conditions: initial tumor volume and MNs administration among others, to obtain a more homogeneous distribution of nanoparticles and improve the therapeutic effect of MFH based on MNs.

## Conclusions

This study reports a multimodal approach to assess biological properties of MNPs extracted from magnetotactic bacteria. A clearly detectable increase of temperature has been recorded by exposing MNs to an AMF. In addition, the temperature increase showed a good linear relation when measured in samples with increasing concentrations of MNs. When investigated *in vivo* by MRI, chains of our MNs are easily detectable if injected directly in the living tissue due to their iron content. This suggests a possible use of these nanoparticles as negative contrast agent or magnetic tracer. This behavior is useful to localize a bolus of MNs injected in a tumor mass: we were able to visualize the site of injection and follow the physiological distribution of nanoparticles within the tumor over time by MR imaging. Moreover, the same modality allows to record the effect of AMF exposures on tumors at different time points. Although we did not observe a reduction of tumor mass, areas of fibrosis and necrosis were visible at microscopic level.

Multimodal approach is a valuable strategy to characterize several aspects of MNs. Many more efforts are needed to further understand key aspects of their interactions with living systems [32], [33], [34], both healthy or not, but these results enforce the hypothesis of a potential, minimally invasive, therapeutic application of nanoparticles-based hyperthermia.
